# Associations of Circadian Rhythms with Cognitive Performance in Patients with Amnestic Mild Cognitive Impairment (aMCI)

**DOI:** 10.3390/jcm15083023

**Published:** 2026-04-15

**Authors:** Seong Jae Kim, Jung Hie Lee, Jae-Won Jang, Minseo Choi, In Bum Suh

**Affiliations:** 1 Department of Psychiatry, College of Medicine, Chosun University, Gwangju 61453, Republic of Korea; kseongjae@hanmail.net; 2 Department of Psychiatry, College of Medicine, Kangwon National University, Chuncheon 24341, Republic of Korea; 3 Department of Psychiatry, Gwanggyo Good Sleep Clinic, Suwon 24341, Republic of Korea; 4 Department of Neurology, College of Medicine, Kangwon National University, Chuncheon 16508, Republic of Korea; jaewon26@gmail.com; 5 Institute of Health Policy and Management, Seoul National University Medical Research Center, Seoul 03080, Republic of Korea; minseochoiknuh@gmail.com; 6 Department of Laboratory Medicine, Sangju Red Cross Hospital, Sangju 37199, Republic of Korea; bloodmd@kangwon.ac.kr

**Keywords:** amnestic mild cognitive impairment (aMCI), sleep–wake timings, rest–activity rhythm (RAR), dim light melatonin onset (DLMO), cognitive performance

## Abstract

**Background/Objectives**: Circadian rhythm disruption is linked to cognitive decline, yet it remains unclear how behavioral and physiological rhythm markers are differently associated with cognition in amnestic mild cognitive impairment (aMCI). The primary aim of this study was to compare sleep–wake timing, rest–activity rhythm (RAR), and dim light melatonin onset (DLMO) between patients with aMCI and cognitively normal controls. Exploratory analyses further examined their associations with domain-specific cognitive performance. **Methods:** Eighteen aMCI patients and 21 cognitively normal controls (NC) enrolled. Cognitive function was assessed using the Korean version of the Consortium to Establish a Registry for Alzheimer’s Disease Neuropsychological Battery (CERAD-K). Participants underwent 5-day actigraphy to assess sleep–wake timing and non-parametric RAR variables, including interdaily stability (IS), intradaily variability (IV), and relative amplitude (RA). DLMO was determined from hourly salivary melatonin samples collected over five hours before sleep onset under dim-light conditions. Group comparisons of circadian markers were conducted as the primary analyses, and generalized linear models were used for exploratory analyses of associations between circadian markers and cognitive outcomes. **Results**: Groups did not significantly differ in sleep–wake timing, RAR parameters and DLMO. Sleep–wake timing variables and DLMO were not significantly associated with cognitive performance. Higher IS was associated with better visuospatial memory and executive function, whereas higher RA was associated with poorer verbal memory among aMCI patients. **Conclusions:** Although sleep–wake timing and melatonin phase did not differ between groups nor predict cognitive performance, higher daily rhythm stability was linked to better non-verbal memory and executive functioning. In contrast, high RA may relate to poorer verbal memory in aMCI, suggesting that elevated RA may not reflect true circadian robustness required for optimal cognition.

## 1. Introduction

Circadian rhythms are intrinsic clocks that coordinate a wide range of behavioral and physiological processes, including the sleep–wake cycle, hormone secretion, and thermoregulation [1,2]. These endogenous rhythms play a pivotal role in maintaining optimal cognitive performance across one’s lifespan, particularly in older adults [3,4]. Disruptions in circadian organization, manifested as weakened rhythmicity or phase misalignment (e.g., delayed or irregular sleep–wake cycles), have been increasingly recognized as contributors to age-related cognitive decline and dementia risk [5,6].

Alzheimer’s disease (AD) is characterized by circadian rhythm disturbances, including irregular sleep–wake cycles and altered melatonin secretion [5,7]. These alterations are thought to arise from the early degeneration of the suprachiasmatic nucleus (SCN), the brain’s master circadian pacemaker. Accumulating evidence also suggests that impairments in cognitive domains, such as memory, attention, and executive function, often coincide with circadian dysregulation [5,6]. Circadian rhythm disruption may further increase the vulnerability of the hippocampus and prefrontal cortex, which are among the regions that are affected earlier in AD [5]. These brain areas depend on circadian regulation to maintain the daily neural activity that underlies learning, memory, and executive functioning [8,9,10].

Rest–activity rhythm (RAR) has emerged as a sensitive behavioral indicator of circadian rhythmicity [6,11]. It reflects the temporal structure and consistency of daily activity patterns and serves as a behavioral output of the central circadian regulatory system. Reduced rhythmicity or amplitude in RAR, indicative of circadian dysregulation, has been linked to AD-related pathology such as amyloid-β accumulation and cognitive decline [8]. Recent studies have shown that weaker or fragmented RAR is associated with tau and amyloid pathology, as well as poorer cognition [11,12], highlighting its potential as an early biomarker in AD. Moreover, community-dwelling older adults with diminished RAR regularity tend to exhibit prefrontal dysfunction, which leads to deficits in executive function, attention, and working memory [6,8].

Beyond RAR, sleep–wake timing variables, including bedtime, midsleep time, and wake-up time, serve as behavioral indicators of the circadian phase, reflecting how well an individual’s sleep–wake pattern is entrained to environmental and social cues [13]. While recent studies have begun to recognize variability in sleep–wake patterns as a potential contributor to cognitive impairment, most prior research has focused primarily on variability in sleep duration or efficiency [14] rather than on these timing variables. However, studies that directly examine alterations in sleep–wake timing and their association with cognitive performance remain scarce.

Dim light melatonin onset (DLMO) is the most reliable physiological marker of the endogenous circadian phase and directly reflects the timing of the central biological clock. In AD, DLMO is often delayed, and its amplitude is reduced, indicating a pronounced disruption of circadian regulation. In contrast, findings on mild cognitive impairment (MCI) are mixed, with some studies reporting delayed or reduced DLMO and others showing preserved patterns [15]. Given that melatonin secretion is closely linked to sleep regulation and cognitive processing, alterations in DLMO in MCI may represent early manifestations of circadian misalignment preceding clinical dementia. Nevertheless, systematic investigations on the relationship between DLMO and cognitive decline in patients with MCI remain limited [7].

Amnestic mild cognitive impairment (aMCI) is widely recognized as the prodromal stage of AD, and is primarily characterized by memory dysfunction [16,17]. Compared to the non-amnestic subtype, aMCI shows a stronger association with AD-related neurodegeneration [18,19]. The multi-domain subtype of aMCI reflects a more extensive cognitive decline and is considered a more sensitive indicator of early AD pathology than the single-domain subtype, which is characterized by isolated memory deficits [20].

Given the close interplay between circadian regulation and cognitive functioning, illustrating circadian parameters in patients with aMCI may provide valuable insights into early neurodegenerative mechanisms. Although circadian disruption has been reported in general MCI and AD populations, evidence specific to amnestic aMCI, particularly the multi-domain subtype, remains limited. Because multi-domain aMCI is characterized by broader cognitive deficits beyond memory, it represents a clinically important subgroup. Few studies have simultaneously assessed behavioral circadian markers, such as sleep–wake timing and rest–activity rhythm (RAR), together with the physiological phase marker dim light melatonin onset (DLMO) in patients with aMCI [21]. This lack of an integrated approach limits understanding of how multidimensional circadian dysfunction relates to domain-specific cognitive impairment in aMCI.

Therefore, this study evaluated both behavioral indicators, sleep–wake timing and RAR, and the physiological phase marker, DLMO, to comprehensively characterize the circadian function in aMCI. The primary aim of this study was to compare circadian markers—including sleep–wake timing variables, dim light melatonin onset (DLMO), and non-parametric rest–activity rhythm variables—between patients with aMCI and cognitively normal controls. We then conducted exploratory analyses to examine the associations between these circadian markers and domain-specific cognitive performance in executive function, attention, and memory. We hypothesized that patients with aMCI would exhibit greater circadian disruption than cognitively normal controls, and that greater circadian disruption would be associated with poorer cognitive performance.

Using this integrated approach, the present study aimed to examine the clinical relevance of behavioral and physiological circadian alterations in aMCI.

## 2. Materials and Methods

This study was conducted as part of the MCRL project and approved by the Institutional Review Board of Kangwon National University Hospital (Chuncheon, Republic of Korea; approval no. KNUH-A-2020-06-005). Written informed consent was obtained from all participants and their legally authorized representatives before enrollment. All the procedures were performed in accordance with the ethical standards of the Declaration of Helsinki.

### 2.1. Participants and Clinical Assessment

Participants aged ≥50 years were consecutively recruited from July 2020 to December 2021 from the Memory Clinic at Kangwon National University Hospital and two regional public Dementia Care Centers. A screening interview was conducted to obtain baseline information on participants’ medical histories, including comorbid conditions and current medications. All participants underwent clinical and neuropsychological assessments conducted by trained psychiatrists and neuropsychologists.

The diagnosis of aMCI was made by a psychiatrist or neurologist according to Petersen’s criteria [16], which requires subjective or objective memory impairment, the absence of dementia, and a performance of 1.5 standard deviations below the age-, sex-, and education-adjusted norms in at least one memory domain. To exclude dementia, functional status was assessed clinically by psychiatrists or neurologists, who confirmed preserved daily functioning and the absence of functional decline sufficient for a diagnosis of dementia. The aMCI group included patients with both single-domain aMCI, defined as isolated memory impairment, and multi-domain aMCI, defined as memory impairment plus impairment in at least one additional non-memory domain.

Participants were excluded if they met any of the following criteria: (1) current substance-related disorders, depressive disorders, or other psychiatric conditions diagnosed according to the Diagnostic and Statistical Manual of Mental Disorders, Fifth Edition (DSM-5) [21]; (2) medical or behavioral conditions known to affect circadian rhythms (e.g., shift work, recent jet lag); (3) serious medical illnesses, including liver cirrhosis, chronic pulmonary disease, cancer, uncontrolled diabetes, or uncontrolled hypertension; (4) current use of medications that influence sleep or circadian rhythms (e.g., melatonin, Aspirin^®^, hypnotics); (5) suspected or diagnosed primary sleep disorders.

Primary sleep disorders were screened through clinical interviews and questionnaire-based assessment, including the Sleep Disorders Questionnaire subscales for narcolepsy (NAR; ≥30 for males, ≥31 for females), sleep apnea (SA; ≥36 for males, ≥32 for females), and periodic limb movements (PLM; ≥21 for both males and females), using sex-specific cutoff values [22].

Eighteen patients with aMCI (76.56 ± 6.1 years; M: F = 7:11) and 21 NCs (70.43 ± 6.97 years; M: F = 10:11) were finally selected for the study.

### 2.2. Procedures

#### 2.2.1. Cognitive and Clinical Assessments

Cognitive function was assessed using the Korean version of the Consortium to Establish a Registry for Alzheimer’s Disease Neuropsychological Battery (CERAD-K) [23]. The battery included the following subtests: verbal fluency (VF) and the Boston Naming Test (BNT) to evaluate language function, Word List Memory (WLM) to measure immediate verbal memory, Word List Recall (WLR1) to assess delayed verbal memory, Word List Recognition (WLR2) to evaluate verbal recognition memory, Constructional Praxis (CP) to assess visuospatial abilities, Constructional Recall (CR) to evaluate delayed visuospatial memory, Trail-Making Test parts A and B (TMT-A and TMT-B) to assess attention, processing speed, and cognitive flexibility, and the Stroop Color-Word Test (SCWT) to measure inhibitory control and executive functioning. Z-scores adjusted for age, sex, and education were calculated for each test to allow standardized comparisons across participants, allowing standardized comparisons across participants while partially accounting for demographic influences. For clinical interpretation, a Z-score of ≤−1.5 was considered to indicate the threshold for objective cognitive impairment.

In addition, depressive symptoms, chronotype, daytime sleepiness, sleep quality, and insomnia severity were assessed using the Korean versions of the Geriatric Depression Scale (GDS-K), Morningness–Eveningness Questionnaire (MEQ-K), Epworth Sleepiness Scale (KESS), Pittsburgh Sleep Quality Index (PSQI), and Insomnia Severity Index (ISI), respectively. The GDS-K is scored from 0 to 30, with higher scores indicating more severe depressive symptoms. The MEQ-K ranges from 16 to 86, with higher scores indicating greater morning preference. The KESS ranges from 0 to 24, with higher scores indicating greater daytime sleepiness. The PSQI ranges from 0 to 21, with higher scores indicating poorer sleep quality. The ISI ranges from 0 to 28, with higher scores indicating more severe insomnia symptoms.

#### 2.2.2. Actigraphy Monitoring and Circadian Rhythm Analysis

To assess sleep–wake timing and rest–activity patterns, participants wore a wrist-worn actigraphy device (Actiwatch 2; Philips Respironics, USA) continuously on their non-dominant wrist for five consecutive days at home. They were instructed to remove the device only while bathing or in cases of significant discomfort. Sleep diaries were used concurrently to support the actigraphy-based estimates. Consistent with our previously established protocol [24], sleep diaries were used to define the intended sleep period, whereas actigraphy provided objective rest–activity data. Data without movement and/or light signal for 1 h or more were treated as missing data. When diary entries were inconsistent with actigraphy signals, actigraphy data and prespecified quality-control rules were prioritized. When compliance with wearing the Actiwatch was problematic (i.e., no movement nor light signal for 4 h/day or more), data for those days were discarded.

Sleep–wake timing variables, including bedtime, sleep onset, wake time, and midsleep time, were derived based on the sleep period, which was defined as the time from lights off to lights on, according to the participants’ sleep diaries.

Non-parametric circadian rhythm variables were computed using the “NarACT” package in R [23]. Inter-daily stability (IS), intra-daily variability (IV), and relative amplitude (RA) were derived as indices of circadian rhythm regularity and fragmentation. IS reflects the consistency of activity patterns across days, calculated as the ratio of the variance of the average 24 h pattern to the total variance, while IV indicates the fragmentation of rest–activity patterns within a day, calculated as the ratio of the mean squares of the difference between consecutive hours to the total variance. RA represents the amplitude of circadian rhythmicity, calculated as (M10 − L5)/(M10 + L5), where M10 and L5 denote the average activity levels during the most active 10 h and least active 5 h periods, respectively.

Actigraphy data were originally recorded in 1 min epochs using Actiware-Sleep software (version 6.0.2; Philips Respironics, Murrysville, PA, USA). A wake-threshold value of 40 activity counts was applied, as this setting has been reported to provide an optimal balance between sensitivity and specificity for detecting sleep and wake compared with polysomnography. For non-parametric RAR analysis, epoch-level daily rest–activity data were averaged and aggregated into hourly bins. Data quality was evaluated before analysis. Days with more than four consecutive hours of missing data were excluded, and participants with at least three valid recording days were included in the final circadian rhythm analysis. Some participants were excluded from the sleep parameter and RAR analyses because of non-compliance with actigraphy procedures or an insufficient number of valid recording days. Accordingly, sleep parameter analyses were conducted in participants with sufficient actigraphy and sleep diary data (aMCI = 17, NC = 20), whereas RAR analyses were restricted to those with at least three valid actigraphy recording days after quality control (aMCI = 13, NC = 19).

#### 2.2.3. Melatonin Sampling and Dim Light Melatonin Onset (DLMO)

To assess the circadian phase, salivary melatonin samples were collected at the participants’ homes under dim light conditions (<15 lx). Participants were provided with a desk lamp in which the light intensity had been set as less than 15 lux. Sampling occurred every hour over a five-hour period, beginning three hours prior to each participant’s habitual sleep onset time and ending one hour afterward, as determined through sleep diaries and actigraphy. Saliva samples were stored at −80 °C and later analyzed using enzyme-linked immunosorbent assay (ELISA) kits (Direct Saliva ELISA kit, Bühlmann Laboratories AG, Schönenbuch, Switzerland). DLMO was defined as the time at which the melatonin concentration first exceeded 3 pg/mL and remained above this threshold for at least two consecutive measurements. Reduced sample size in the DLMO analysis was mainly related to difficulties with home-based salivary melatonin collection, including reduced compliance with repeated sampling, blood contamination in some samples, and possible unintended light exposure despite use of the provided lamp. Of the 27 participants from whom salivary samples were obtained, two were excluded from the DLMO analysis because one showed a zigzag melatonin profile and the other had melatonin values of 0 pg/mL across the entire sampling window. DLMO was directly determined in 9 participants (aMCI, *n* = 2; NC, *n* = 7), while it was estimated in the remaining 16 participants according to predefined rules when direct determination was not possible within the sampling window. If melatonin levels exceeded the 3 pg/mL threshold at only a single time point and the profile was considered compatible with a plausible ascending pattern, DLMO was estimated by linear interpolation between the last value below threshold and the first value above threshold. If all samples remained below 3 pg/mL, DLMO was estimated as 30 min after the final sampling time. Conversely, if all samples were already above 3 pg/mL, DLMO was estimated as 30 min before the first sampling time [25]. Sensitivity analyses using alternative thresholds (e.g., 2 pg/mL) were not performed.

### 2.3. Statistical Analysis

Neuropsychological test scores were standardized into z-scores using age-, sex-, and education-adjusted normative data. Group comparisons of demographic characteristics and cognitive scores were performed using independent *t*-tests or chi-squared tests, as appropriate.

Generalized Linear Models (GLMs) were conducted to examine the effects of sleep–wake timing variables (e.g., sleep onset, wake time, midsleep time), non-parametric circadian rhythm variables (interdaily stability [IS], intradaily variability [IV], and relative amplitude [RA]), and DLMO on cognitive performance. Because the dependent variables were continuous z-scores, the models were specified using a Gaussian distribution with an identity link. In each model, the group status (aMCI vs. NC), circadian variables of interest, and their interaction terms were included as predictors. Additionally, model diagnostics were assessed by visual inspection of residual plots, Q–Q plots, and influential observations, and no major violations were identified.

No a priori sample size or power calculation was performed. Given the modest sample size and the inclusion of multiple predictors, these present analyses should be interpreted as exploratory and may have been underpowered to detect small-to-moderate associations. Accordingly, no formal adjustment for multiple comparisons was applied.

All statistical analyses were performed using SPSS software (version 18.0; SPSS Inc., Chicago, IL, USA). A two-tailed *p*-value < 0.05 was considered statistically significant.

## 3. Results

### 3.1. Demographic and Clinical Characteristics

The aMCI group was significantly older than the NC group (*p* = 0.006) and had significantly higher GDS-K scores (*p* = 0.009). No statistically significant differences were detected in the MEQ-K, KESS, PSQI, or ISI scores between the two groups (Table 1).

### 3.2. Neurocognitive Functions

Compared with the NC group, the aMCI group showed significantly lower performance in most neurocognitive domains, including the VF, BNT, MMSE-KC, WLM, CP, WLR1, WLR2, CR, and SCWT (all *p* < 0.05). No statistically significant differences were detected in the TMT-A and TMT-B (Table 2).

### 3.3. Sleep–Wake Timing, Rest–Activity Rhythms, and Melatonin Rhythms

Although 39 participants (18 aMCI, 21 NC) were initially enrolled, not all participants contributed usable data to every analysis because of incomplete protocol completion or failure to meet predefined data quality criteria. Specifically, sleep parameter analyses were conducted in 37 participants with sufficient actigraphy and sleep diary data (17 aMCI, 20 NC), whereas RAR analyses were restricted to 32 participants with at least three valid days of actigraphy recording after quality control (13 aMCI, 19 NC). For melatonin assessment, 27 participants attempted the home-based salivary melatonin sampling protocol. After excluding two participants because of inadequate or failed sampling, 25 participants were included in the final analysis (9 with directly calculated DLMO and 16 with estimated DLMO).

After controlling for age, no statistically significant differences were detected in sleep timing variables, including bedtime, sleep onset, wake time, and midsleep time, nor were there significantly differences between the aMCI and NC groups (all *p* > 0.05). Additionally, no statistically significant differences were detected between the groups in IS, IV, or RA. Furthermore, no statistically significant differences in DLMO were detected between the aMCI and NC groups after controlling for age (*p* = 0.125) (Table 3).

### 3.4. Effects of the Sleep–Wake Timing Variables on the Cognitive Functions

No significant main or interaction effects of sleep–wake timing variables on cognitive performance were found in the GLMs (Table 4).

### 3.5. Effects of the RAR Variables on the Cognitive Functions

**Figure 1:**
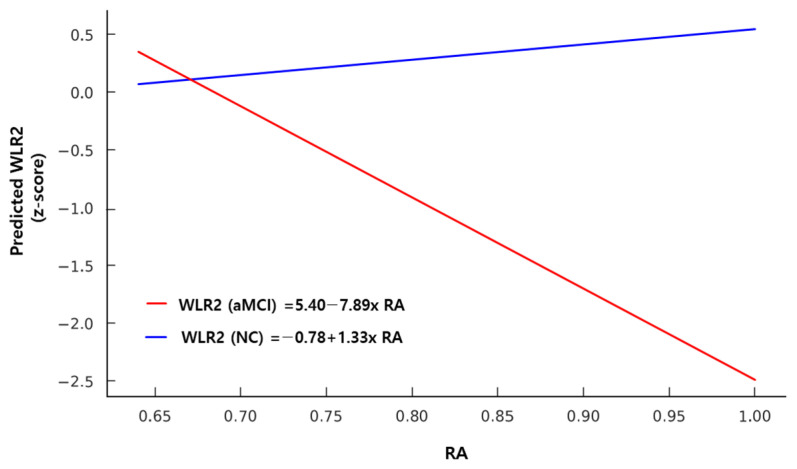
Predicted marginal effects of relative amplitude (RA) on Word List Recognition (WLR2) scores, separately for the aMCI and NC groups. A significant group × RA interaction effect was observed (*p* = 0.01). The red line represents the generalized linear model for the aMCI group, and the blue line represents the model for the NC group.

A significant main effect of IS was found on both constructional recall (CR) (β = 2.61, 95% CI [1.15, 5.07], *p* = 0.027) and the SCWT (β = 2.03, 95% CI [0.22, 4.28], *p* = 0.049), indicating that greater day-to-day rhythm regularity was associated with better performance in visuospatial memory and executive function. Additionally, a significant interaction effect between group and RA was observed for WLR2, indicating that higher RA was associated with lower WLR2 performance in the aMCI group (β = −8.51, 95% CI [−16.07, −0.95], *p* = 0.010), but not in the NC group (Table 5) (Figure 1).

### 3.6. Effects of the DLMO on the Cognitive Functions

DLMO was not significantly associated with any cognitive variable, and no significant group by DLMO interactions were found (Table 6).

## 4. Discussion

The primary aim of this study was to compare circadian markers between patients with aMCI and cognitively normal controls, as well as to explore the associations between these markers and cognitive performance. Overall, our findings did not support the hypothesis of between-group differences in circadian markers, but they did support the hypothesis that circadian alterations are associated with poorer performance in specific cognitive domains.

### 4.1. Clinical and Neurocognitive Characteristics of aMCI

Patients with aMCI showed higher depression scores (GDS-K) than normal controls, which is consistent with previous findings that depressive symptoms are common among individuals with cognitive decline [26,27]. This elevated level of depression is generally understood to reflect emotional distress arising from the self-awareness of cognitive decline [28]. Depressive symptoms, sleep–wake disturbances, and cognitive impairment may arise from overlapping neurobiological mechanisms related to circadian dysregulation, which affects the regulation of sleep–wake cycles, neuroendocrine rhythms, and synaptic plasticity that collectively support mood and cognitive function [5,29]. Therefore, whether depression in MCI is a confounding factor or a distinct clinical phenotype that reflects the underlying pathophysiology of cognitive impairment remains to be determined.

### 4.2. Circadian Rhythm Profiles in aMCI

In this study, patients with aMCI exhibited significantly lower performance than NC across most neurocognitive domains, except for the TMT. This broad impairment, particularly in memory and language, likely reflects the relatively high proportion of the multi-domain subtype in our aMCI group (*n* = 18; 8 single-domain, 10 multi-domain). Previous studies have identified memory and language deficits as early markers of progression from aMCI to AD [30,31], suggesting that our sample may reflect prominent early cognitive vulnerability. Importantly, this subtype composition should be considered when interpreting the associations between circadian variables and cognition, as these associations may differ between single-domain and multi-domain aMCI. Although the present sample size did not permit a reliable subgroup comparison, this possibility warrants further investigation in larger studies.

In this study, no significant differences were found in sleep–wake timing parameters, including bedtime, sleep onset, wake time, and midsleep time, between the aMCI and NC groups (Table 3). These findings suggest that, unlike patients with AD who often show delayed sleep onset or irregular wake times, individuals with aMCI may not exhibit clear alterations in sleep–wake timing [7,32]. Despite the lack of significant differences, these findings suggest that circadian sleep–wake timing remains relatively preserved in early cognitive decline. It has received less attention than sleep quality or architecture in previous studies [33,34].

Similarly, no significant group differences were found in RAR metrics, including IS, IV, and RA, between the aMCI and NC groups (Table 3). These results indicated that daily activity patterns were largely maintained during the early stages of cognitive decline, with no clear signs of circadian disruption. We have previously shown in a related MCI cohort [35] that RAR parameters remain largely preserved, which is consistent with the current findings in aMCI. In contrast, patients with AD typically exhibit disrupted RAR profiles, characterized by reduced regularity, increased fragmentation, and diminished amplitude [6,32,36].

DLMO, a biological marker of the circadian phase, did not differ significantly between the aMCI and NC groups (Table 3). Although the aMCI group showed a numerically earlier mean DLMO, these findings suggest that prominent shifts in the melatonin rhythm may not occur during the prodromal stage of cognitive decline. Previous findings in individuals with MCI remain limited and inconsistent, with some studies reporting advanced DLMO compared to controls [21]. In contrast, studies on patients with AD have frequently reported a delayed melatonin phase, reflecting neurodegenerative changes in the SCN and associated circadian dysregulation [37]. Taken together, the lack of group differences in sleep–wake timing, RAR, and DLMO in this study does not provide clear evidence of overt circadian disruption in patients with aMCI. In this modest sample, behavioral measures such as sleep–wake timing and activity rhythm, as well as DLMO, did not differ significantly between groups. However, these findings should be interpreted cautiously and should not be taken as definitive evidence of preserved circadian organization or phase regulation.

### 4.3. Associations Between Circadian Rhythm Parameters and Cognitive Function

No significant associations were found between the sleep- wake timing variables and cognitive performance (Table 4). While disruptions in sleep–wake timing are well documented in AD and linked to melatonin dysregulation or SCN dysfunction [38], such alterations are not yet evident in aMCI, possibly reflecting relatively preserved circadian regulation at this stage. Since prior MCI studies have mainly focused on sleep quality and sleep architecture rather than timing-related variables [39], these null findings should be interpreted with caution, given the limited evidence on the role of sleep timing in aMCI.

Our findings revealed that greater IS, which reflects the consistency of daily activity rhythms, was significantly associated with better performance in visuospatial memory (CR) and executive function tests (SCWT) (Table 5). This is consistent with previous reports showing that more stable circadian rhythms are associated with preserved cognitive performance and a lower risk of cognitive decline in older adults [11,40]. Given that nonverbal memory and executive control rely on the coordinated function of the hippocampus, occipitoparietal association areas, and prefrontal networks, which are influenced by the circadian modulation of sleep-dependent memory consolidation and cortical arousal [3,9,41], this association may reflect the beneficial effects of stable daily rhythmicity in supporting memory integration and higher-order cognitive control. Stable circadian organization likely facilitates optimal synchronization between the sleep–wake cycle and cortical arousal rhythms, thereby enhancing neural efficiency in tasks that require memory retrieval and executive coordination. Together, these findings suggest that maintaining regular daily routines and consistent activity–rest patterns may help preserve nonverbal memory and executive functioning in individuals with aMCI. From a clinical perspective, these findings suggest that behavioral strategies aimed at enhancing day-to-day circadian stability may be relevant for older adults with aMCI. In particular, maintaining structured daily routines and optimizing daytime light exposure may help support more stable sleep–wake patterns and daily activity rhythms. Such behavioral and environmental approaches may represent practical chronotherapeutic strategies for future investigation in patients with aMCI.

In contrast, the RA, representing the contrast between daytime and nighttime activity levels, showed a significant group-by-RA interaction in relation to cognitive performance, indicating an unexpected negative association in the aMCI group (Figure 1). Specifically, in participants with aMCI, higher RA was associated with poorer verbal memory scores. This finding contrasts with previous studies that reported that greater circadian regularity or amplitude is generally linked to better cognitive performance in older adults [11,40]. A high RA may indicate stronger circadian rhythmicity, but an excessively elevated RA does not necessarily reflect restorative sleep [42]. One possible explanation is that, in some individuals with aMCI, higher RA may reflect reduced behavioral flexibility rather than truly robust circadian function; however, this interpretation remains hypothetical and is not directly supported by the present data. Therefore, this finding should be considered preliminary and interpreted cautiously, and future dedicated studies are needed before any firm conclusions can be drawn.

In summary, while sleep–wake timing variables were not significantly associated with cognitive performance, greater stability of daily activity rhythms (IS) showed meaningful associations with visuospatial and executive functions, suggesting the beneficial effects of maintaining consistent daily patterns. In contrast, the unexpected negative association between the RA and verbal memory implies that excessively rigid or dysregulated activity rhythms may adversely affect cognitive performance in individuals with aMCI. Overall, these findings suggest that the stability and adaptive flexibility of circadian rhythms, rather than the timing of sleep itself, may play a pivotal role in maintaining cognitive function during aMCI.

In this study, general linear model (GLM) analyses revealed no significant main effects of DLMO on cognitive performance and no significant interactions between DLMO and the diagnostic group (aMCI vs. NC) across the cognitive domains (Table 6). Although previous studies have reported delayed or attenuated melatonin rhythms in AD and in preclinical at-risk older adults [43,44], only a few studies have examined DLMO in MCI, with inconsistent findings. For example, Naismith et al. [21] observed a delayed melatonin onset, whereas Nous et al. (2021) [45] reported an advanced phase. These findings suggest that DLMO alterations may not be directly related to cognitive performance during the prodromal (aMCI) stage. However, given the limited sample size, these findings should be interpreted with caution. Furthermore, methodological limitations—specifically our narrow 5 h sampling window with 1 h resolution—may not have captured the exact DLMO. This may have increased the risk of circadian phase misclassification, particularly in individuals with particularly early or late melatonin onset, and may have obscured subtle associations between DLMO and cognitive performance. Accordingly, the null findings should not be interpreted as evidence of no relationship.

This study had several limitations. First, the relatively small sample size limited the statistical power, and subtle effects may have gone undetected. As this was a small-sample study with multiple predictors and interaction terms, the analyses should be interpreted as exploratory, and parameter estimates may have been underpowered to detect small-to-moderate associations. In addition, because multiple statistical tests were conducted and *p*-values were not adjusted for multiplicity, the findings—particularly those near the conventional significance threshold—should be interpreted as hypothesis-generating rather than confirmatory. Second, some participants were excluded from the sleep parameter and RAR analyses because of incomplete actigraphy/sleep diary data or insufficient valid actigraphy recording days, which may have introduced selection bias. In addition, the actigraphy the monitoring period was limited to five consecutive days, and the RAR analysis was based on hourly aggregated rest–activity data, which may have restricted the ability to capture broader day-to-day variability in rest–activity rhythms and more subtle rest–activity rhythm variations. Third, the study population consisted of older Korean adults recruited from a single province, which may limit the generalizability of the findings to other regional or ethnic populations. Fourth, owing to the cross-sectional study design, the observed associations should be interpreted as correlational rather than causal. Fifth, because salivary samples were collected at home, strict dim-light conditions (<15 lx) could not be objectively verified. This, together with the relatively narrow 5 h sampling window, may have introduced misclassification of circadian phase, particularly in participants with very early or very late melatonin onset. Sixth, primary sleep disorders were screened through clinical interviews and questionnaires, but objective assessments such as polysomnography or home sleep apnea testing were not systematically performed. Therefore, undetected sleep-disordered breathing or other sleep-related conditions may have influenced the findings. Finally, although we used age-, sex-, and education-adjusted z-scores for cognitive outcomes, residual confounding cannot be excluded. In group comparisons of circadian variables, some analyses were adjusted for age, but depressive symptoms (GDS-K) were not additionally controlled. Moreover, in the generalized linear models examining associations between circadian markers and cognitive performance, age and GDS-K were not included as covariates because of concerns about overfitting and reduced statistical power in this relatively small sample. In addition, because formal comparisons between included and excluded participants were not performed, potential attrition bias could not be fully assessed.

## 5. Conclusions

Despite these limitations, this study provided novel insights into the multidimensional relationship between circadian rhythmicity and cognitive function in individuals with aMCI. To the best of our knowledge, this is the first study to concurrently evaluate sleep–wake timing, actigraphy-derived RAR indices, and biological phase markers (DLMO) within the same cohort. By integrating behavioral and physiological indicators of circadian organization, our approach extends previous single-marker studies and highlights the functional relevance of rhythmic stability and adaptability. Importantly, the associations of greater stability of daily activity rhythms (IS) with better nonverbal memory and executive performance, and of higher RA with poorer verbal memory, should be considered preliminary but promising findings. These results suggest that stability and adaptive flexibility of circadian rhythms, rather than timing per se or rhythm amplitude alone, may be relevant to cognitive function in aMCI. Larger longitudinal studies are needed to confirm these associations, clarify causal pathways, and explore rhythm-based biomarkers and interventions aimed at preserving cognitive function in populations at risk of dementia.

## Figures and Tables

**Table 1:** Demographic and clinical characteristics in the aMCI (*n* = 18) and NC (*n* = 21) groups.

	aMCI Group	NC Group	*p*-Value
Age (years) ^†^	76.56 ± 6.1 *	70.43 ± 6.97	0.006
Gender (M: F) ^‡^	7:11	10:11	0.584
Education(years) ^†^	7.78 ± 5.11	10.29 ± 2.99	0.065
GDS-K	13.33 ± 7.96 *	8.52 ± 4.52	0.009
MEQ-K	64.17 ± 6.82	61.95 ± 8.98	0.564
KESS	6.89 ± 4.48	7.33 ± 4.41	0.496
PSQI	3.28 ± 2.61	3.95 ± 2.8	0.517
ISI	6.78 ± 4.62	7.9 ± 5.53	0.447

*: *p* < 0.01 (^†^ Independent *t*-test, ^‡^ X^2^ test or ANCOVA controlling for age). aMCI: amnestic mild cognitive impairment, NC: normal controls; GDS-K: Korean version of the Geriatric Depression Scale; MEQ-K: Korean version of the Morningness–Eveningness Questionnaire; KESS: Korean version of the Epworth Sleepiness Scale; PSQI: Pittsburgh Sleep Quality Index; ISI: Insomnia Severity Index.

**Table 2:** Neurocognitive functions ^†^ in the aMCI (*n* = 18) and NC (*n* = 21) groups.

	aMCI Group	NC Group	*p* Value
VF	−0.82 ± 0.54 **	0.47 ± 1.13	0.001
BNT	−0.33 ± 1.2 *	0.65 ± 0.7	0.011
MMSE-KC	−0.95 ± 0.88 *	−0.09 ± 1.14	0.032
WLM	−0.94 ± 0.78 **	1.01 ± 1.07	0.000
CP	−0.42 ± 1.46 *	0.44 ± 0.64	0.027
WLR1	−1.44 ± 0.83 **	0.34 ± 0.78	0.000
WLR2	−1.77 ± 1.49 **	0.4 ± 0.53	0.000
CR	−1.41 ± 0.62 **	0.56 ± 1.03	0.000
TMT-A	0.18 ± 0.68	0.6 ± 0.79	0.136
TMT-B	0.45 ± 0.84	0.5 ± 1.21	0.699
SCWT	−1.02 ± 0.8 *	0.14 ± 0.81	0.002

*: *p* < 0.05, **: *p* < 0.01 (independent *t*-test). ^†^: Given values are z-scores adjusted for age, sex, and education. aMCI: amnestic mild cognitive impairment, NC: normal controls; VF: verbal fluency, BNT: Boston naming test, WLM: word list memory, WLR1: word list recall, CP: constructional praxis, WLR2: word list recognition, CR: constructional recall, TMT-A,-B: trail making test-A-B: trail making test-B, SCWT: stroop color-word test.

**Table 3:** Sleep–wake timing, rest–activity rhythms, and melatonin rhythm variables in the aMCI and NC groups.

	aMCI Group	NC Group	*p*-Value
Sleep timings	(*n* = 17)	(*n* = 20)	
Bedtime (h:m)	22:00 ± 1.5	22:49 ± 1.0	0.244
Sleep onset (h:m)	22:20 ± 1.4	22:59 ± 0.9	0.503
Wake time (h:m)	05:55 ± 1.1	06:38 ± 1.2	0.171
Midsleep time (h:m)	03:47± 0.8	03:50 ± 0.6	0.522
Rest–activity rhythm	(*n* = 15)	(*n* = 19)	
IS	0.61 ± 0.15	0.56 ± 0.16	0.164
IV	0.85 ± 0.33	0.75 ± 0.23	0.496
RA	0.89 ± 0.84	0.90 ± 0.92	0.347
Melatonin rhythm	(*n* = 9)	(*n* = 16)	
DLMO (h:m)	21:06 ± 2.7	22:41 ± 1.9	0.125

ANCOVA controlling for age. aMCI: amnestic mild cognitive impairment, NC: normal controls; IS: interdaily stability, IV: intradaily variability, RA: relative amplitude; DLMO: Dim light melatonin onset.

**Table 4:** Main effects of sleep–wake variables and group by them interactions on the VF, WLM, WLR1, WLR2, CR and SCWT (*n* = 37: aMCI = 17, NC = 20).

	*SO*	*Group by SO*	*WT*	*Group by WT*	*MST*	*Group by MST*
VF						
B (95% CI)	0.004(−0.45, 0.46)	0.079(−0.48, 0.64)	0.222(−0.13, 0.57)	−0.169(−0.70, 0.36)	0.443(−0.26, 1.14)	−0.534(−1.46, 0.39)
Partial η^2^	0.003	0.002	0.030	0.012	0.026	0.002
WLM						
B (95% CI)	0.002(−0.47, 0.47)	−0.062(−64, 0.52)	0.059 (−0.31, 0.43)	0.007(−0.56, 0.57)	0.116(−0.62, 0.85)	0.068(−0.91, 1.05)
Partial η^2^	0.001	0.001	0.006	0.000	0.000	0.001
WLR1						
B (95% CI)	0.005(−0.40, 0.40)	0.029 (−0.46, 0.52)	0.190(−0.12, 0.50)	−0.136(−0.60, 0.33)	0.377(−0.24, 0.99)	−0.378(−1.19, 0.44)
Partial η^2^	0.001	0.000	0.031	0.010	0.020	0.004
WLR2						
B (95% CI)	0.083(−0.40, 0.57)	0.179(−0.42, 0.78)	0.070(−0.32, 0.46)	0.161(−0.43, 0.75)	0.038(−0.75, 0.82)	−0.254(−1.29, 0.78)
Partial η^2^	0.038	0.010	0.029	0.009	0.054	0.017
CR						
B (95% CI)	−0.078(−0.51, 0.35)	0.050(−0.48, 0.58)	0.196(−0.14, 0.53)	−0.269(−0.78, 0.24)	0.492(−0.17, 1.15)	−0.524(−1.40, 0.35)
Partial η^2^	0.005	0.001	0.007	0.032	0.001	0.012
SCWT						
B (95% CI)	0.103(−0.28, 0.49)	0.102(−0.37, 0.58)	0.176(−0.13, 0.48)	−0.274(−0.74, 0.19)	0.228(−0.36, 0.82)	−0.700(−1.48, 0.81)
Partial η^2^	0.048	0.006	0.003	0.040	0.045	0.002

Generalized linear model. Values are presented as unstandardized coefficients (B) with 95% confidence intervals and Partial η^2^ for effect sizes. Higher coefficients indicate greater change in the cognitive z-score per one-unit increase in the corresponding predictor. aMCI: amnestic mild cognitive impairment, NC: normal controls; SO: sleep onset, WT: wake time, MST: midsleep time; VF: verbal fluency, BNT: Boston naming test, WLM: word list memory, WLR1: word list recall, WLR2: word list recognition, CR: Constructional recall, SCWT: stroop color-word test.

**Table 5:** Main effects of nonparametric RAR variables and group by them interactions on the VF, WLM, WLR1, WLR2, CR and SCWT (*n* = 32: aMCI = 13, NC = 19).

	*IS*	*Group by IS*	*IV*	*Group by IV*	*RA*	*Group by RA*
VF						
B (95% CI)	1.572(−1.10, 4.24)	−0.422(−4.67, 3.82)	0.971(−1.04, 2.98)	−0.933(−3.58, 1.71)	−1.469(−6.55, 3.61)	3.261(−5.35, 11.87)
Partial η^2^	0.003	0.040	0.016	0.084	0.001	0.026
WLM						
B (95% CI)	1.760(−0.97, 4.49)	1.145(−3.20, 5.49)	−1.315(−3.35, 0.72)	1.947(−0.73, 4.63)	5.549(0.70, 10.40)	−5.765(−13.98, 2.45)
Partial η^2^	0.129	0.009	0.025	0.037	0.076	0.042
WLR1						
B (95% CI)	1.949(−0.36, 4.26)	−2.353 (−6.03, 1.32)	0.455(−1.17, 2.08)	−0.568(−2.71, 1.57)	1.556(−2.38, 5.49)	−4.593(−11.26, 2.07)
Partial η^2^	0.022	0.050	0.000	0.023	0.001	0.036
WLR2						
B (95% CI)	−0.074(−2.78, 2.63)	−3.402(−7.71, 0.91)	0.207(−1.81, 2.22)	0.029(−2.62, 2.68)	1.328(−3.13, 5.79)	−8.510 ** (−16.07, −0.95)
Partial η^2^	0.080	0.074	0.014	0.004	0.096	0.173
CR						
B (95% CI)	2.61 ** (0.15, 5.07)	−2.541(−6.45, 1.37)	−0.380(−2.15, 1.39)	1.264(−1.06, 3.59)	0.198(−4.30, 4.70)	−2.803(−10.42, 4.81)
Partial η^2^	0.057	0.051	0.001	0.007	0.001	0.003
SCWT						
B (95% CI)	2.03 * (−0.22, 4.28)	−2.87(−6.46, 0.73)	0.269(−1.42, 1.96)	−0.392(−2.61, 1.83)	2.877(−0.96, 6.71)	−1.789(−8.29, 4.71)
Partial η^2^	0.016	0.084	0.001	0.004	0.050	0.011

*: *p* < 0.05, ** *p* < 0.01 (Generalized linear model). Values are presented as unstandardized coefficients (B) with 95% confidence intervals and Partial η^2^ for effect sizes. Higher coefficients indicate greater change in the cognitive z-score per one-unit increase in the corresponding predictor. For practical interpretation, higher IS indicates greater rhythm stability, higher IV indicates greater fragmentation, and higher RA indicates stronger rest–activity contrast; IS and RA range from 0 to 1. aMCI: amnestic mild cognitive impairment, NC: normal controls; IS: interdaily stability, IV: intradaily variability, RA: relative amplitude; VF: verbal fluency, BNT: Boston naming test, WLM: word list memory, WLR1: word list recall, WLR2: word list recognition, CR: Constructional recall, SCWT: stroop color-word test.

**Table 6:** Main effects of DLMO and grouped by their interactions on the VF, WLM, WLR1, WLR2, CR and SCWT (*n* = 25: aMCI = 9, NC = 16).

	*DLMO*	*Group by DLMO*
VF		
B (95% CI)	−0.018 (−0.29, 0.26)	−0.037 (−0.42, 0.34)
Partial η^2^	0.006	0.001
WLM		
B (95% CI)	0.153 (−0.09, 0.40)	−0.311 (−0.27, 0.24)
Partial η^2^	0.000	0.114
WLR1		
B (95% CI)	0.074 (−0.11, 0.26)	−0.016 (−0.21, 0.29)
Partial η^2^	0.040	0.001
WLR2		
B (95% CI)	−0.089 (−0.27, 0.09)	0.041 (−0.45, 0.46)
Partial η^2^	0.045	0.004
CR		
B (95% CI)	0.150 (−0.10, 0.40)	−0.143 (−0.49, 0.21)
Partial η^2^	0.031	0.025
SCWT		
B (95% CI)	−0.053 (−0.26, 0.16)	0.070 (−0.23, 0.37)
Partial η^2^	0.002	0.009

Generalized linear model. Values are presented as unstandardized coefficients (B) with 95% confidence intervals and Partial η^2^ for effect sizes. Higher coefficients indicate greater change in the cognitive z-score per one-unit increase in DLMO. For practical interpretation, higher DLMO values indicate a later circadian phase. aMCI: amnestic mild cognitive impairment, NC: normal controls, DLMO: Dim light melatonin onset; VF: verbal fluency, BNT: Boston naming test, WLM: word list memory, WLR1: word list recall, WLR2: word list recognition, CR: constructional recall, SCWT: stroop color-word test.

## Data Availability

The data supporting the findings of this study are available within the article.

## Abbreviations

The following abbreviations are used in this manuscript:

aMCI	amnestic mild cognitive impairment
NC	normal controls
GDS-K	Korean version of the Geriatric Depression Scale
MEQ-K	Korean version of the Morningness-Eveningness Questionnaire
KESS	Korean version of the Epworth Sleepiness Scale
PSQI	Pittsburgh Sleep Quality Index
ISI	Insomnia Severity Index
CERAD-K	Korean version of the Consortium to Establish a Registry for Alzheimer’s Disease assessment battery
MMSE-KC	Mini-Mental State Examination in the Korean version of the CERAD Assessment Packet
VF	verbal fluency
BNT	Boston Naming Test
WLM	Word List Memory
WLR1	Word List Recall
WLR2	Word List Recognition
CP	constructional praxis
CR	constructional recall
TMT-A	Trail Making Test-A
TMT-B	Trail Making Test-B
SCWT	Stroop Color-Word Test
BT	bedtime
SO	sleep onset
WT	wake time
MST	midsleep time
IS	interdaily stability
IV	intradaily variability
RA	relative amplitude
DLMO	dim light melatonin onset
RAR	rest–activity rhythm
SCN	suprachiasmatic nucleus
DSM-5	Diagnostic and Statistical Manual of Mental Disorders, Fifth Edition
ELISA	enzyme-linked immunosorbent assay
GLM	generalized linear model
AD	Alzheimer’s disease
